# Intermittent Fasting Ameliorates Testicular Damage via Oxidative Stress Modulation in a Genetic Absence Epilepsy Rat Model †

**DOI:** 10.3390/ijms27083619

**Published:** 2026-04-18

**Authors:** Damla Gökçeoğlu Kayalı, Hatice Maraş, Aybüke Çilingir, Ahmet Anıl Keskin, Çağan Yardımcı, Fatma Beyza Aykurt, Eda Nur Arslan, Bircan Kolbaşı-Erkan, Zarife Nigar Özdemir-Kumral, Ozlem Tugce Cilingir-Kaya

**Affiliations:** 1 Department of Histology and Embryology, School of Medicine, Istanbul Atlas University, 34408 Istanbul, Türkiye; 2 Department of Histology and Embryology, School of Medicine, Marmara University, 34854 Istanbul, Türkiye; htcisn@gmail.com; 3 School of Medicine, Marmara University, 34854 Istanbul, Türkiye; aybukecilingir1@icloud.com (A.Ç.); aanilkeskin60@gmail.com (A.A.K.); caganyardimci@gmail.com (Ç.Y.); fatmabeyzaaykurt@gmail.com (F.B.A.); aedanur57@gmail.com (E.N.A.); 4 Department of Histology and Embryology, School of Medicine, Istanbul Medipol University, 34815 Istanbul, Türkiye; bkolbasi@medipol.edu.tr; 5 Department of Physiology, School of Medicine, Marmara University, 34854 Istanbul, Türkiye; zarifeozdemir@gmail.com

**Keywords:** intermittent fasting, epilepsy, oxidative stress, testis, PIWI proteins, DAZL protein, caspase 3

## Abstract

Epilepsy is associated with impaired reproductive function and testicular pathologies. Intermittent fasting (IF) is a nonpharmacological metabolic intervention with anti-inflammatory and antioxidant effects. This study investigated the protective effects of IF on testicular damage in a genetic absence epilepsy rat model (GAERS), focusing on histomorphology, oxidative stress parameters, and hormonal profiles. Testicular tissues from Wistar control (WC), Wistar + IF (WIF), GAERS control (GC), and GAERS + IF (GIF) groups (total *n* = 20; 5 rats per group) were evaluated using hematoxylin and eosin and Periodic Acid–Schiff staining. Apoptosis and spermatogenic cell integrity were assessed using caspase-3, P-element-induced wimpy testis (PIWI), and Deleted in Azoospermia-Like (DAZL) immunohistochemistry. Johnsen’s score, seminiferous tubule diameter, and epithelial thickness were quantified. Oxidative stress markers, including catalase, malondialdehyde, glutathione, myeloperoxidase, and superoxide dismutase, were measured using spectrophotometric methods, and serum testosterone, luteinizing hormone (LH), and follicle-stimulating hormone (FSH) levels were determined using ELISA kits. The GC group showed significantly reduced Johnsen scores, tubular diameters, and epithelial thickness, along with disrupted basement membrane integrity and increased caspase-3 immunoreactivity. IF significantly improved histological parameters, restored basement membrane integrity, reduced apoptosis, and increased PIWI and DAZL expression in the GIF group. IF also ameliorated oxidative stress and elevated reproductive hormone levels, indicating positive modulation of the hypothalamic–pituitary–gonadal axis. In conclusion, IF reduces oxidative stress and preserves seminiferous tubules and hormonal function in genetic absence epilepsy, highlighting its potential as a supportive nonpharmacological approach to protect male reproductive health.

## 1. Introduction

Epilepsy is a chronic neurological disorder that affects approximately 1% of the global population. Although its exact pathogenesis remains unclear, accumulating evidence suggests that oxidative stress, neuroinflammation, and disturbances in neurotransmitter signaling contribute to epileptogenesis [1,2,3]. Absence epilepsy is a subtype of generalized epilepsy characterized by spike–wave discharges and typically begins during childhood. Nevertheless, experimental models, such as Genetic Absence Epilepsy Rats from Strasbourg (GAERS), are widely used to investigate the mechanisms and systemic consequences of absence epilepsy. These models allow the study of the long-term pathological effects of epileptic activity, including those observed in adult animals [4]. Studies have shown that individuals with absence epilepsy have a higher prevalence of comorbid conditions such as attention deficits, anxiety, and hyperactivity [5]. Individuals with epilepsy often experience progressive neurological deterioration, psychosocial disorders and systemic complications [1,6,7]. These factors not only contribute to neurological dysfunction but also affect peripheral systems, including the reproductive system. Male patients with epilepsy frequently exhibit reproductive and hormonal disturbances such as hypogonadism, reduced fertility, and testicular atrophy [8,9]. Noninvasive therapeutic strategies that can be implemented early are needed to mitigate the systemic effects of childhood absence epilepsy, particularly on the reproductive system.

Intermittent fasting (IF), a noninvasive dietary intervention involving alternating fasting and feeding periods, has emerged as a potent modulator of cellular metabolism.

It enhances antioxidant defense mechanisms, decreases ROS production, and induces autophagy, thereby offering protection against metabolic and neurodegenerative diseases [10]. Although the beneficial effects of IF on the central nervous system are well documented, its potential role in alleviating epilepsy-induced reproductive dysfunction remains unclear.

Previous studies have reported that IF can influence male reproductive physiology by modulating oxidative stress, metabolic pathways, and hormonal regulation. Experimental studies in rodents have shown that IF reduces lipid peroxidation, enhances antioxidant enzyme activity, and preserves testicular architecture under metabolic or toxic stress. Moreover, IF affects the hypothalamic–pituitary–gonadal (HPG) axis activity and spermatogenesis through redox-sensitive signaling pathways. However, the effects of IF on reproductive pathology associated with epilepsy, particularly in genetic absence epilepsy models such as GAERS, remain largely unexplored [11,12,13,14].

Fertility rates are lower among individuals with epilepsy than in the general population, and reproductive endocrine disorders are more prevalent in this group [15,16,17,18,19]. Approximately 20% of men with epilepsy report decreased libido or potency, and over 90% exhibit abnormal semen parameters, including reduced sperm count, impaired motility, and morphological abnormalities [20,21,22,23]. Experimental epilepsy models have also demonstrated testicular abnormalities, including atrophic seminiferous tubules, vacuolization, disrupted basal membranes, impaired intercellular junctions, and increased apoptosis [24]. The GAERS strain is a well-established model of absence epilepsy [25,26]. Given the high oxidative stress burden associated with epilepsy, IF may offer a protective strategy against testicular degeneration and hormonal imbalance [27].

Therefore, the present study aimed to investigate the effects of intermittent fasting on testicular damage in GAERS rats by evaluating oxidative stress markers, histopathological alterations, germ cell maturation and reproductive hormone levels. We hypothesized that IF would ameliorate testicular pathology through its antioxidant and antiapoptotic effects.

## 2. Results

The body weights were measured at the beginning and at the end of the IF. Body weight increased in all groups; this increase is significant in the WIF group compared to the WC group (Figure S1). 

**Figure 1:**
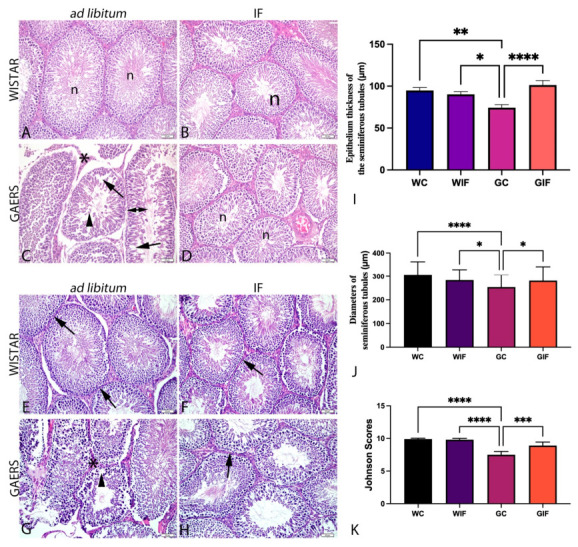
Representative micrographs of the experimental groups. (**A**–**D**): H&E staining ((**A**): WC, (**B**): WIF, (**C**): GC, and (**D**): GIF). n: normal seminiferous tubules with intact germinal epithelium; arrow: vacuolization; arrowhead: desquamation; double-headed arrow: thinning of the seminiferous epithelium; asterisk (*): disruption in the interstitial area. Scale bar: 50 µm. (**E**–**H**): PAS staining ((**E**): WC, (**F**): WIF, (**G**): GC, (**H**): GIF). Arrow: PAS-positive basement membrane; asterisk (*): irregular basement membrane; arrowhead: disorganization of the seminiferous tubule epithelium. Scale bar: 50 µm. Quantitative analyses are shown in panels (**I**–**K**): (**I**) seminiferous epithelial thickness, (**J**) tubule diameter, and (**K**) Johnsen score. Data are presented as mean ± SEM. Statistical analysis was performed using two-way ANOVA to assess the main effects of Epilepsy and Diet, as well as their interaction, followed by Tukey’s post-hoc test. * *p* < 0.05, ** *p* < 0.01, *** *p* < 0.001, **** *p* < 0.0001.

Microscopic evaluation of hematoxylin and eosin (H&E) stained sections revealed normal seminiferous tubules with regular germinal epithelia in the WC, WIF, and GIF groups. Vacuole formation, immature cell/desquamation in the lumen of the seminiferous tubules, and edema formation were detected in the GC group, and the interstitial area largely lost its integrity. Epithelial thinning was also observed in the GC group (Figure 1A–D).

Periodic-acid Schiff (PAS)-positive basement membrane structure with regular morphology was observed in the seminiferous tubules of the WC and WIF groups. In contrast, the GC group displayed irregular basement membranes with undulations in some seminiferous tubules. In the GIF group, many seminiferous tubules exhibited a PAS-positive basement membrane and regular morphology (Figure 1E–H).

Two-way ANOVA revealed significant main effects of epilepsy and diet, as well as their interactions, on the evaluated parameters. The Johnsen score was significantly lower in the GC group than in the WC, WIF, and GIF groups (*p* < 0.0001, *p* < 0.0001, and *p* < 0.001, respectively). Furthermore, seminiferous tubule thickness and diameter were lower in the GC group than in the Wistar group. However, this decrease showed a trend towards control levels with IF treatment (Figure 1K).

**Figure 2:**
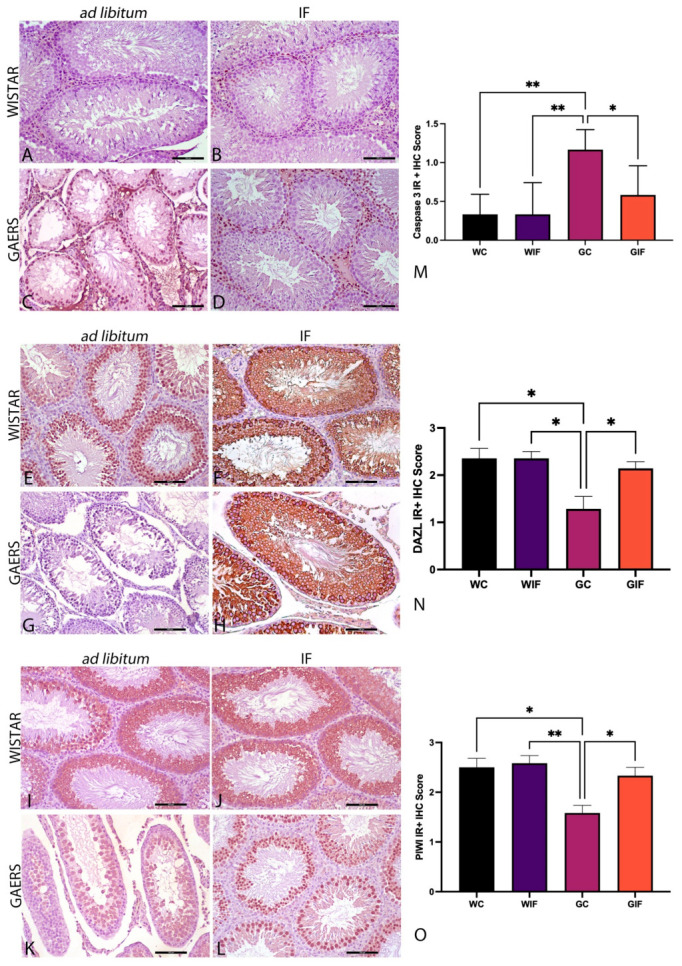
Representative micrographs showing caspase-3, DAZL, and PIWI immunoreactivities in the experimental groups. Caspase-3 immunoreactivity is shown in (**A**–**D**) ((**A**): WC, (**B**): WIF, (**C**): GC, (**D**): GIF), DAZL-positive cells in (**E**–**H**) ((**E**): WC, (**F**): WIF, (**G**): GC, (**H**): GIF), and PIWI immunoreactivity in (**I**–**L**) ((**I**): WC, (**J**): WIF, (**K**): GC, (**L**): GIF). Scale bar: 100 µm. Quantitative analyses of immunoreactive cell density are presented in panels (**M**–**O**). (**M**) Caspase-3-immunoreactive cell density, (**N**) DAZL-immunoreactive cell density, and (**O**) PIWI-immunoreactive cell density in the testis. Data are presented as mean ± SEM. Statistical analysis was performed using two-way ANOVA to assess the main effects of Epilepsy and Diet, as well as their interaction, followed by Tukey’s post hoc test. * *p* < 0.05, ** *p* < 0.01.

Microscopic evaluation revealed a small number of caspase 3-positive cells in the seminiferous tubules of the WC, WIF, and GIF groups. Conversely, an increased number of caspase 3-positive cells was observed in the seminiferous tubules of the GC group (Figure 2A–D).

A large number of deletion of azoospermia-like (DAZL)-positive cells were observed in the seminiferous tubules of the WC, WIF, and GIF groups, whereas only a small number of DAZL-positive cells were observed in the seminiferous tubules of the GC group (Figure 2E–H).

A large number of P-element-induced wimpy testis (PIWI)-positive cells were observed in the seminiferous tubules of the WC, WIF, and GIF groups, whereas significantly fewer PIWI-positive cells were detected in the GC group. Quantitative analysis confirmed a significant decrease in PIWI-positive cell density in the GC group compared to the WC and WIF groups, whereas partial restoration was observed in the GIF group (Figure 2I–L).

**Figure 3:**
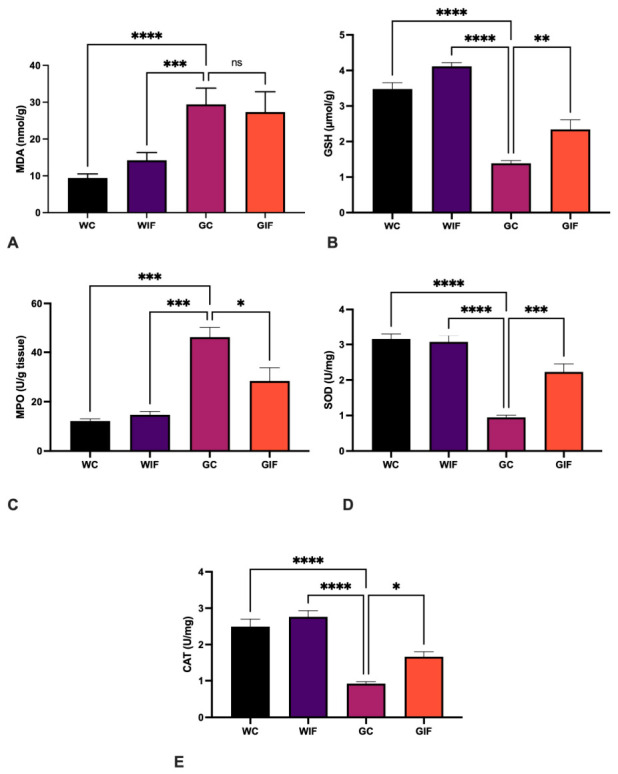
Oxidative stress marker levels in the tissue samples. Quantitative analysis of MDA (**A**), GSH (**B**), MPO (**C**), SOD (**D**), and CAT (**E**) levels. Data are presented as mean ± SEM. Statistical analysis was performed using two-way ANOVA to assess the main effects of Epilepsy and Diet, as well as their interaction, followed by Tukey’s post-hoc test. ns: non-spesific, * *p* < 0.05, ** *p* < 0.01, *** *p* < 0.001, **** *p* < 0.0001.

Two-way ANOVA revealed significant main effects of epilepsy and diet on the oxidative stress parameters. Catalase (CAT) levels were significantly lower in the GC group than in the WIF and WC groups, whereas the reduction was less significant than that in the GIF group (A) (*p* < 0.0001, *p* < 0.0001, and *p* < 0.05, respectively). The superoxide dismutase (SOD) levels were significantly lower in the GC group than in the WIF and WC groups, whereas the reduction was less significant than that in the GIF group (B) (*p* < 0.0001, *p* < 0.0001, and *p* < 0.001, respectively). Glutathione (GSH) levels were significantly lower in the GC group than in the WIF and WC groups, whereas the reduction was less significant than that in the GIF group (C) (*p* < 0.0001, *p* < 0.0001, and *p* < 0.05, respectively). Myeloperoxidase (MPO) levels were significantly higher in the GC group than in the WIF and WC groups, whereas the increase was less significant than that in the GIF group (D) (*p* < 0.001, *p* < 0.0001, and *p* < 0.05, respectively). Malondialdehyde (MDA) levels were significantly higher in the GC group than in the WIF and WC groups (E) (*p* < 0.001 and *p* < 0.0001, respectively) (Figure 3).

**Figure 4:**
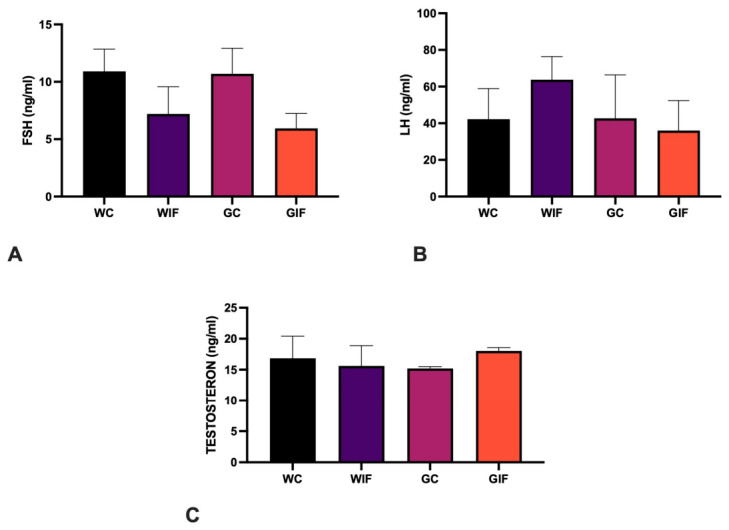
Serum hormone levels in the experimental groups. (**A**): Quantitative analysis of serum follicle-stimulating hormone (FSH) levels; (**B**): Quantitative analysis of serum luteinizing hormone (LH) levels; (**C**): Quantitative analysis of serum testosterone levels. Data are presented as mean ± SEM. Statistical analysis was performed using two-way ANOVA to assess the main effects of Epilepsy and Diet, as well as their interaction, followed by Tukey’s post hoc test.

Two-way ANOVA revealed no significant main effects of epilepsy or diet, nor a significant interaction between these factors on serum hormone levels. Analysis of serum gonadotropin and testosterone concentrations revealed minor alterations among the experimental groups. FSH levels showed a modest reduction in the IF group compared to the control group, whereas an apparent elevation was observed in the GC group. However, the GIF group exhibited the most pronounced decline in FSH concentrations (Figure 4A). A similar trend was observed for the LH levels. Although the GC and GIF groups exhibited values comparable to those of the WC group, LH levels were significantly higher in the WIF group (Figure 4B). This suggests that IF upregulates LH levels, which are reduced in epileptic rats. Testosterone levels remained relatively stable between the control and IF groups but showed a substantial decrease in the GC group, which became even more pronounced following IF (GIF group) (Figure 4C). This implies a potential suppressive effect of absence epilepsy on testosterone levels, which was only partially counteracted by IF treatment.

## 3. Discussion

IF has been extensively documented to have systemic benefits, including metabolic regulation, antioxidant activity, and neuroprotection [10,28]. While previous studies have primarily focused on these broad physiological advantages across various organ systems, the present study provides novel evidence highlighting the protective role of IF against testicular damage in the context of epilepsy. Specifically, we demonstrated that IF ameliorates epilepsy-induced reproductive pathology, addressing a critical gap in the literature [29]. Consistent with earlier findings reporting systemic oxidative stress in epileptic models [30], we observed significant elevations in the oxidative stress biomarkers, MDA and MPO, in the GAERS group. Notably, IF attenuated these increases while concurrently restoring key antioxidant markers, such as SOD and GSH. This restoration of oxidative balance suggests that IF may exert its protective effects, at least in part, through the modulation of intracellular redox pathways, a mechanism that requires further investigation.

Using a targeted experimental approach, this study examined the restorative effects of IF on testicular damage in GAERS rats, with particular emphasis on oxidative stress markers, histopathological alterations, and germ cell maturation. A one-month IF regimen was implemented to assess its potential protective role in epilepsy-induced testicular dysfunction. Although IF improved oxidative stress markers and histological parameters in epileptic rats, testosterone levels were not restored to control levels in the GAERS + IF group and showed a further decrease compared with those in the GC group. These findings suggest that while IF exerts protective effects on testicular morphology and oxidative balance, its endocrine effects in epileptic conditions may be limited or context-dependent [31].

Isojarvi reported separation and vacuolization of the germinal epithelium, seminiferous tubule degeneration, edema, and a significant decrease in sperm in the lumen of the seminiferous tubules in the testes of rats with epilepsy [16]. Consistently, our findings confirmed that the absence of epilepsy results in testicular degeneration, as evidenced by reduced seminiferous tubule diameter, epithelial thinning, and impaired germ cell maturation. Histopathological analyses further revealed that IF significantly improved seminiferous architecture in epileptic rats. The presence of PAS-positive basement membranes, normalized seminiferous tubule diameter, and improved Johnsen scores, a widely accepted method for evaluating germ cell maturation, in the GIF group supports this conclusion. Importantly, caspase-3 expression, an indicator of apoptosis, was significantly reduced after IF treatment. These findings align with the known anti-apoptotic effects of IF in other models and indicate the involvement of apoptosis-regulating pathways, such as caspase signaling and autophagy, in mediating tissue protection [8,24]. These outcomes are consistent with reports that IF promotes cellular resilience by enhancing autophagy and antioxidant defense [10].

PIWI proteins belong to a family of proteins essential for RNA silencing and gene regulation. These proteins form ribonucleoprotein complexes that bind to Piwi-interacting RNAs (piRNAs) and function primarily in germ cells to suppress transposons and regulate gene expression [32]. DAZL is another critical protein that governs germ cell development during the embryonic stage. It facilitates gene expression by stabilizing mRNAs and promoting its translation within germ cells [33]. The reduced number of PIWI- and DAZL-positive cells in the GC group indicates disrupted spermatogenesis, reinforcing the notion that epilepsy adversely affects germ cell maturation. Notably, IF alleviated these disruptions in the GIF group, as evidenced by improved Johnsen scores and increased PIWI and DAZL expressions. These findings suggest that IF may help restore or preserve spermatogenesis in epileptic animals, potentially by modulating oxidative stress and inflammatory pathways, both of which contribute to germ cell damage. However, molecular validation of these pathways (e.g., piRNA pathway integrity and DAZL-regulated mRNA stabilization) remains an important step.

In this study, the levels of the oxidative stress markers MDA, GSH, MPO, and SOD provided important insights into the impact of IF on testicular oxidative damage. Previous studies comparing ketogenic diets and IF in adult male rats reported significantly lower MDA and MPO levels and elevated SOD, CAT, and GSH levels in IF-treated animals, indicating a reduction in oxidative stress [11,12,34,35]. Consistent with these findings, the GC group in our study exhibited increased MDA and MPO levels and decreased GSH and SOD levels, reflecting the elevated oxidative stress typical of epileptic conditions. Notably, the IF regimen significantly reversed these changes in the GIF group. These biochemical improvements coincided with reduced testicular damage, consistent with earlier reports highlighting the cytoprotective effects of IF on testicular cells. Further studies are needed to investigate the underlying mechanisms, with particular emphasis on autophagy and neuroinflammatory regulation of the disease.

A key contribution of this study was the integration of hormonal analysis with morphological and biochemical data into the literature. The GAERS group exhibited reduced testosterone and intermediate LH levels, confirming disruption of the HPG axis secondary to epilepsy. IF alone elevated testosterone and LH levels in control rats, which is consistent with reports that fasting enhances GnRH pulsatility and Leydig cell function [36]. However, these hormonal improvements were blunted in the GIF group, suggesting that the neuropathophysiology of epilepsy may limit the endocrine benefits of IF. The interplay between neuroinflammation, glucocorticoid signaling, and HPG dysregulation likely contributes to this outcome and should be explored in future studies.

Importantly, this nuanced hormonal response contrasts with the literature indicating reduced testosterone levels in healthy males undergoing IF [30]. Acknowledging these discrepancies, further studies should explore the effects of IF on reproductive hormones under various neuroinflammatory and metabolic conditions.

In the present study, we examined the effects of IF on reproductive hormones, including FSH, LH, and testosterone, in GAERS rats. In control animals, IF alone tended to increase LH levels, consistent with reports that IF stimulates the hypothalamic–pituitary–gonadal (HPG) axis by enhancing GnRH secretion and subsequent LH and FSH release. This likely promotes testosterone production by Leydig cells and supports optimal testicular functions. Conversely, the GAERS group displayed decreased testosterone and intermediate LH levels, indicating epilepsy-related disruption of hormonal regulation. Epileptic seizures impair hypothalamic function and suppress GnRH release, which may lead to gonadotropin deficiency and reduced testosterone synthesis [29,36,37,38]

Our results highlight that epilepsy impairs male reproductive function and increases testicular oxidative stress. However, IF ameliorated these adverse effects by reducing oxidative markers and improving histopathological outcomes. While the existing literature has separately addressed epilepsy-related sexual dysfunction and neuroinflammation [39], our study uniquely integrated these domains, demonstrating that IF can act as a holistic modulator. Although IF increased LH and testosterone levels in healthy rats, epilepsy suppressed these hormones in epileptic rats. The IF + GAERS group exhibited diminished hormonal improvements, highlighting the complex interplay between chronic neurological diseases and metabolic interventions.

These findings suggest that IF may be a promising nonpharmacological strategy for mitigating reproductive dysfunction in epilepsy [40]. In contrast to pharmacological treatments, IF is a holistic approach that promotes systemic metabolism and neuroprotection [41]. However, clinical trials are necessary to assess the safety, feasibility, and optimal fasting protocols in humans. Such research may inform dietary strategies to alleviate epilepsy-related reproductive complications and improve the quality of life.

Although our results provide several novel insights, certain limitations should be recognized. First, the sample size (*n* = 5 per group) was determined a priori using G*Power 3.1 analysis based on an estimated large effect size (f = 0.76) to ensure statistical power while adhering to the 3R principles of animal ethics. While this was sufficient for our primary histological and biochemical endpoints, a larger cohort may be needed for more subtle hormonal variations. Second, the intermittent fasting regimen was assessed for four weeks; additional studies with longer intervals are required to evaluate its long-term effects. Although our results indicate significant improvements in oxidative stress and histopathology, the underlying molecular mechanisms remain to be elucidated in future studies. Finally, this study focused on the GAERS model, a specific genetic form of absence epilepsy. While this model allows for the investigation of chronic, spontaneous seizure activity, the findings may not be directly generalizable to chemical induction models, such as PTZ- or KA-induced epilepsy.

## 4. Materials and Methods

All experimental protocols were approved by the Local Ethics Committee of Marmara University Animal Experiments (protocol no. 71.2023). All procedures were performed in accordance with the institutional guidelines for animal care and use.

### 4.1. Experimental Model

**Figure 5:**
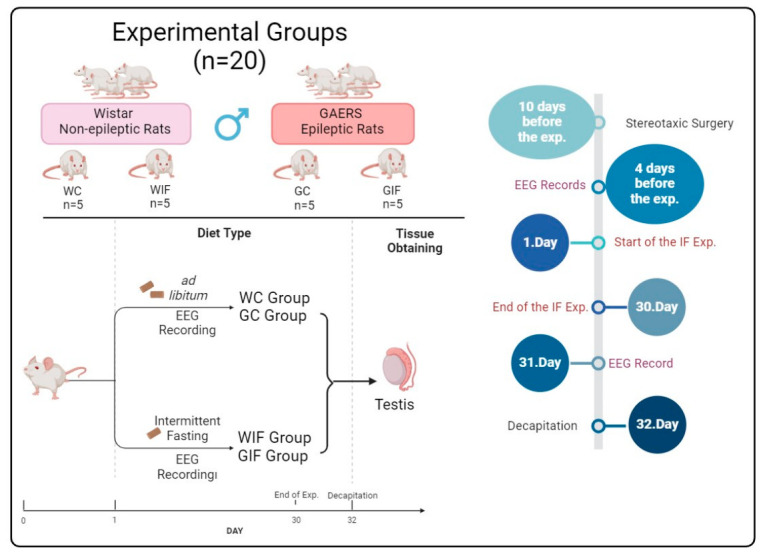
Experimental groups and the graphical abstract.

Four experimental groups were established: Wistar control (WC), Wistar + IF (WIF), GAERS control (GC), and GAERS + IF (GIF), with five animals in each group (*n* = 5) (Figure 5). Eight-week-old adult male rats aged 8 weeks were used in the experiments. The initial body weights were 120–135 g for GAERS rats and 160–185 g for Wistar albino rats. The rats were maintained under standard laboratory conditions with a humidity level of 45–55%, temperature of 18–24 °C, and a 12 h light/dark cycle. Adult male rats with established reproductive activity were selected to evaluate their reproductive function.

**Figure 6:**
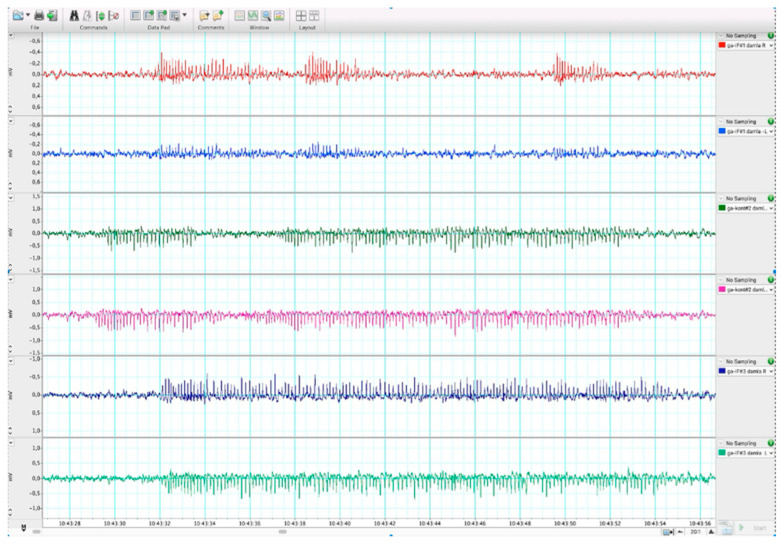
EEG recordings.

Animals in the IF groups were subjected to an alternate-day fasting (ADF) protocol (24 h feeding/24 h fasting) (Figure 5). To confirm epilepsy in GAERS rats, EEG recordings were performed, and spike–wave discharges lasting longer than 5 s were accepted as the criteria for absence epilepsy. Recordings were obtained bilaterally from the frontoparietal cortex three hours after electrode implantation (Figure 6). The animals were randomly assigned to the experimental groups using a computer-generated randomization sequence. Histological evaluations were performed by two independent observers blinded to group allocation. In addition, biochemical and hormonal analyses were conducted using coded samples to minimize observer bias.

All animals were euthanized in the morning during the light phase (09:00–11:00) to minimize circadian variation. Tissue and blood samples from the IF groups were collected at the end of the fasting period to maintain consistency with the fasting protocol and comparable metabolic conditions. At the end of the experimental period, the rats were euthanized under deep anesthesia using a ketamine (100 mg/kg, i.p.) and xylazine (10 mg/kg, i.p.) cocktail.

Blood samples were collected via cardiac puncture and centrifuged at 4 °C and 3000 rpm for 10 min to obtain the serum. The right testis was surgically excised and processed for histological evaluation of the seminiferous tubules. Briefly, the tissues were fixed in 10% neutral buffered formalin for approximately 48 h and dehydrated using a graded ethanol series (70%, 80%, 96%, and 100%). After dehydration, the tissues were cleared with xylene and embedded in paraffin using the standard protocols. The left testis was dissected and divided into three parts for the biochemical evaluation of oxidative stress parameters. Tissue samples intended for MDA, GSH, MPO, and SOD assays were stored at −80 °C until spectrophotometric analysis.

### 4.2. Histochemical Analyses

Using a rotary microtome (Leica RM2125 RTS, Wetzlar, Germany), 4–5 µm thick sections were obtained from paraffin-embedded tissue blocks. The sections were stained with H&E for general histopathological evaluation and with PAS staining to assess the integrity of the basal membrane. Histopathological scoring was performed according to established criteria for assessing edema, inflammation, and cellular degeneration. Three non-consecutive testicular sections were analyzed for each animal. In each section, the seminiferous tubule diameter was measured from 20 randomly selected round tubules, and Johnsen scoring was performed for all tubules present in the analyzed sections. Caspase-3 immunoreactivity was evaluated to determine the levels of apoptosis by quantitatively counting positively stained cells. Two independent observers, blinded to the experimental groups, evaluated the tissues, and any discrepancies were resolved by consensus.

Morphological measurements were performed on hematoxylin and eosin (H&E)-stained testicular sections. The mean seminiferous tubule diameter (MSTD) was measured as an indicator of testicular damage. Tubule diameters were measured using an ocular micrometer at 100× magnification under a light microscope, with ten randomly selected round tubules from different preparations for each animal [42].

The thickness of the seminiferous epithelium was assessed by calculating the average spermatogenic epithelial thickness at five randomly selected points [43]. Testicular damage was evaluated using Johnsen’s scoring system (Table 1) [44,45]. According to this system, all tubules in the sections were examined and scored on a scale of 1–10 based on the level of germ cell maturation.

### 4.3. Immunohistochemical Analyses

Sections obtained from fixed paraffin-embedded tissue blocks were incubated overnight at 37 °C in an oven. The sections were deparaffinized by immersion in xylene (2 × 10 min), followed by rehydration in absolute and 96% ethanol (2 × 10 min each). Endogenous peroxidase activity was blocked by incubating the sections with 3% hydrogen peroxide in methanol for 10 min. After rinsing with distilled water, antigen retrieval was performed by heating the sections in citrate buffer (pH 6.0) in a microwave oven (200 W) for 20 min. Primary antibodies against PIWI (Invitrogen, Carlsbad, CA, USA, PA5-17034; 1:50), DAZL (Invitrogen, MA5-32708), and Caspase-3 (Abcam, Cambridge, UK, ab92552; 1:50) were applied, and the sections were incubated overnight at +4 °C. The following day, the sections were washed twice with phosphate-buffered saline (PBS) for 5 min each and incubated with a biotinylated secondary antibody for 20 min. After an additional wash with PBS, the sections were incubated with streptavidin–peroxidase for 20 min, followed by a final rinse with PBS. Color development was achieved by applying 3,3′-diaminobenzidine (DAB) chromogen for 2 min. The sections were counterstained with Mayer’s hematoxylin for 1 min and rinsed with tap water. Finally, the slides were dehydrated in 96% ethanol (2 × 10 min) and mounted with Entellan.

### 4.4. Determination of Oxidative Stress Markers

Oxidative stress markers were analyzed using spectrophotometric assays, and serum reproductive hormones (FSH, LH, and testosterone) were measured using commercial ELISA kits. The levels of oxidative stress markers, including MDA, GSH, MPO, and SOD, were determined using classical spectrophotometric methods, as described previously.

#### 4.4.1. Determination of Malondialdehyde (MDA)

Tissue MDA levels were determined according to the method described by Beuge and Aust [46]. For the assay, 10% tissue homogenates (prepared for GSH determination) were mixed with 1 mL of 0.375% thiobarbituric acid (TBA) and incubated in a boiling water bath for 15 min. After cooling to RT, the tubes were centrifuged at 3000 rpm for 10 min. The supernatant was collected, and the absorbance of the resulting chromogen was measured using a spectrophotometer.

#### 4.4.2. Determination of Glutathione (GSH)

Tissue GSH levels were measured using Ellman’s method [17]. After decapitation, the testes were immediately excised, washed with physiological saline, blotted dry using filter paper and weighed. Tissue homogenates (10%) were prepared on ice using a 150 mM KCl solution and a homogenizer (Ika Werke, Staufen, Germany). Subsequently, 0.2 mL of 20% trichloroacetic acid (TCA) was added to 0.4 mL of the 10% homogenate, mixed thoroughly, and centrifuged at 3000 rpm for 15 min. The GSH levels in the supernatant were determined, and the pellets were discarded after the measurement. To the supernatant, 1 mL of 0.3 M Na_2_HPO_4_ and 0.05 mL of Ellman’s reagent were added, mixed, and incubated for 5 min. The absorbance of the yellow complex was measured using spectrophotometry.

#### 4.4.3. Determination of Myeloperoxidase (MPO)

Tissue MPO activity was determined using the Hillegass method [19]. After decapitation, the testicular tissues were immediately excised, washed with physiological saline to remove blood and debris, blotted dry with filter paper, and weighed. Testicular tissue was homogenized in 50 mM K_2_HPO phosphate buffer (pH 6.0) to obtain a 10% homogenate. A 3 mL aliquot of the homogenate was centrifuged at 41,400× *g* at 4 °C for 10 min. The supernatant was discarded, and 3 mL of 0.5% hexadecyltrimethylammonium bromide (HETAB) was added to the pellet, followed by homogenization. The samples were subjected to three freeze–thaw cycles followed by sonication. The mixture was centrifuged again at 41,400× *g* and 4 °C for 10 min, and 0.3 mL of the supernatant was collected for further analysis. Subsequent reactions were performed in a water bath at 37 °C. The color reaction was stopped by adding 0.2 mL of 2% sodium azide to each well. After removal from the water bath and mixing, the samples were left at room temperature (RT) and centrifuged at 3000 rpm for 10 min to separate the supernatant from the precipitate. The supernatant was collected, and its absorbance was measured using a spectrophotometer.

#### 4.4.4. Determination of Superoxide Dismutase (SOD)

SOD activity in the tissue homogenates was measured as previously described [47]. Briefly, measurements were performed in cuvettes containing 2.8 mL of 50 mM potassium phosphate buffer (pH 7.8) with 0.1 mM EDTA, 0.1 mM riboflavin, 0.39 mM potassium phosphate buffer (pH 7.5), 0.1 mL of 6 mM o-dianisidine·2HCl, and 50 or 100 µL of the tissue extract. The cuvettes were illuminated with a 20 W Sylvania Grow Lux fluorescent light positioned 5 cm above and incubated at 37 °C. Absorbance was measured using a Shimadzu UV-02 spectrophotometer. A standard curve was prepared using bovine SOD (S-2515; 3000 U; Sigma-Aldrich, Burlington, MA, USA) as a reference curve. Absorbance readings were recorded, and the net absorbance was calculated.

#### 4.4.5. Determination of Hormone Levels

Serum testosterone, LH, and FSH levels were determined using commercial Rat FSH ELISA Kit (Cat. No. EA0015Ra; BT Laboratory, Jiaxing, China), Rat LH ELISA Kit (Cat. No. EA0013Ra; BT Laboratory, Jiaxing, China) and Rat Testosterone ELISA Kit (Cat. No. EA0023Ra; BT Laboratory, Jiaxing, China.). All assays were performed according to the manufacturer’s guidelines.

Following coagulation, blood samples were centrifuged at 2000× *g* for 10 min to obtain serum, which was stored at −80 °C until analysis. Before the assay, all reagents and serum samples were equilibrated at RT. Standard curves were prepared within the concentration ranges recommended by the manufacturer, and each sample was analyzed in duplicate using an ELISA kit. Absorbance was measured at 450 nm using a microplate reader, and hormone concentrations were calculated based on a four-parameter logistic (4-PL) regression model [48].

### 4.5. Statistical Analysis

GraphPad Prism 10.0 (GraphPad Software, San Diego, CA, USA) was used for the statistical analyses. Two-way ANOVA and Tukey’s post hoc test were used for statistical evaluations. Statistical significance was set at *p* < 0.05. Significance levels indicate comparisons between the GC and WC/WIF groups unless otherwise stated.

## 5. Conclusions

In conclusion, this study provides the first evidence that IF exerts protective effects against testicular damage in a genetic absence epilepsy model by attenuating oxidative stress, preserving seminiferous tubule structure, and promoting germ cell maturation. These findings provide a strong foundation for further investigation of dietary interventions as potential adjunctive therapeutic options for epilepsy-associated reproductive dysfunction. Future studies should aim to elucidate the molecular mechanisms involved, particularly the roles of oxidative stress and neuroinflammation in testicular damage and hormonal dysregulation in epilepsy.

## Figures and Tables

**Table 1:** Modified Johnsen’s testicular biopsy scoring criteria [45].

	Histological Description
**Score 1**	No germ cells or Sertoli cells present
**Score 2**	No germ cells; only Sertoli cells are present
**Score 3**	Germ cells present only in the form of spermatogonia
**Score 4**	Very few spermatocytes are present
**Score 5**	No spermatids or spermatozoa are present; however, many spermatocytes are observed
**Score 6**	Only a few spermatids are present
**Score 7**	No spermatozoa are present; however, many spermatids are observed
**Score 8**	A large number of spermatozoa are present
**Score 9**	A large number of spermatozoa are present, but spermatogenesis is disorganized
**Score 10**	Complete spermatogenesis with well-formed, open seminiferous tubules

## Data Availability

The original contributions presented in this study are included in the article/Supplementary Materials. Further inquiries can be directed to the corresponding authors.

## Supplementary Materials

The following supporting information can be downloaded at: https://www.mdpi.com/article/10.3390/ijms27083619/s1.
