# Supplementary material for: Intermittent Fasting Ameliorates Testicular Damage via Oxidative Stress Modulation in a Genetic Absence Epilepsy Rat Model
Source: Int J Mol Sci. 2026 Apr 18;27(8):3619. doi: 10.3390/ijms27083619 (PMC13115940; doi:10.3390/ijms27083619)
Supplement: Supplementary file 1 [file ijms-27-03619-s001.zip › ijms-4170086-supplementary.pdf]

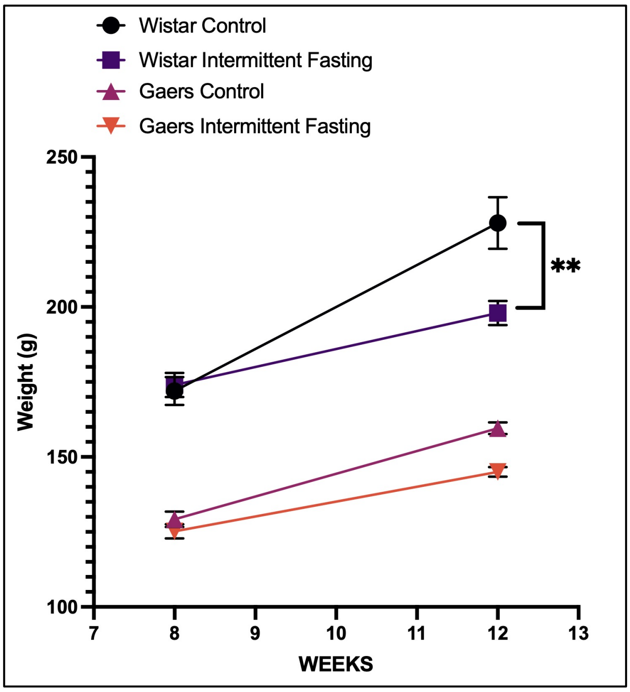

Figure S1. Body weight changes in experimental groups during the study period. Body weights were measured at week 8 (baseline) and week 12 (end of the experiment) in Wistar control (WC), Wistar intermittent fasting (WIF), GAERS control (GC), and GAERS intermittent fasting (GIF) groups. Data are presented as mean ± SD. A significant difference was observed between WC and WIF groups at week 12 (**$p < 0.01$).

Body weight changes during the experimental period are presented in Supplementary Figure S1. While body weight increased in all groups, a significantly lower weight gain was observed in the WIF group compared to the WC group at week 12.
